# TRIM32 Regulates Skeletal Muscle Stem Cell Differentiation and Is Necessary for Normal Adult Muscle Regeneration

**DOI:** 10.1371/journal.pone.0030445

**Published:** 2012-01-27

**Authors:** Sarah Nicklas, Anthony Otto, Xiaoli Wu, Pamela Miller, Sandra Stelzer, Yefei Wen, Shihuan Kuang, Klaus Wrogemann, Ketan Patel, Hao Ding, Jens C. Schwamborn

**Affiliations:** 1 Westfälische Wilhelms-Universität Münster, Zentrum für Molekularbiologie der Entzündung, Institute of Cell Biology, Stem Cell Biology and Regeneration Group, Münster, Germany; 2 Department of Biochemistry and Medical Genetics, University of Manitoba, Winnipeg, Manitoba, Canada; 3 School of Biological Sciences, University of Reading, Whiteknights Campus, Reading, United Kingdom; 4 Department of Animal Sciences, Purdue University, West Lafayette, Indiana, United States of America; McMaster University, Canada

## Abstract

Limb girdle muscular dystrophy type 2H (LGMD2H) is an inherited autosomal recessive disease of skeletal muscle caused by a mutation in the *TRIM32* gene. Currently its pathogenesis is entirely unclear. Typically the regeneration process of adult skeletal muscle during growth or following injury is controlled by a tissue specific stem cell population termed satellite cells. Given that TRIM32 regulates the fate of mammalian neural progenitor cells through controlling their differentiation, we asked whether TRIM32 could also be essential for the regulation of myogenic stem cells. Here we demonstrate for the first time that TRIM32 is expressed in the skeletal muscle stem cell lineage of adult mice, and that in the absence of TRIM32, myogenic differentiation is disrupted. Moreover, we show that the ubiquitin ligase TRIM32 controls this process through the regulation of c-Myc, a similar mechanism to that previously observed in neural progenitors. Importantly we show that loss of TRIM32 function induces a LGMD2H-like phenotype and strongly affects muscle regeneration *in vivo*. Our studies implicate that the loss of TRIM32 results in dysfunctional muscle stem cells which could contribute to the development of LGMD2H.

## Introduction

Limb girdle muscular dystrophies (LGMDs) are a group of inherited disorders that progressively lead to muscle weakness and wasting predominantly in the shoulder and pelvic girdle. Seven types with an autosomal dominant mode of inheritance and 14 autosomal recessive forms have been currently identified [1]. A homozygous mutation in the gene for the protein TRIM32 (D487N) has been linked to LGMD type 2H which was originally identified in the Hutterite population of the North American Prairies [2]. Additional mutations in the *TRIM32* gene leading to LGMD2H have also been found recently in other populations [3], [4], implicating the causative role of TRIM32 in the pathogenesis of LGMD2H. Recently it has been shown that the D489N pathogenic mutation destabilizes the TRIM32 protein, leading to its degradation [5]. Consequently, a potential mechanism of LGMD2H pathology might be the destabilization of mutated TRIM32 protein leading to a null phenotype.TRIM32 belongs to the TRIM-NHL family that is characterized by the presence of an N-terminal RING finger, one or more Zinc-finger-like motifs called B-boxes, a coiled coil region and a C-terminal NHL domain [6]. All of the identified LGMD2H mutations are located in the C-terminal NHL domain [2], [3], [4]. Recently, we have demonstrated that TRIM32 is involved in the regulation of differentiation and self-renewal in neural progenitor cells during mouse embryonic brain development [7] [8]. Additionally, the *Drosophila* orthologs of TRIM32, Brat and the Brat-like protein Mei-P26 control stem cell proliferation in the *Drosophila* nervous system and ovaries, respectively [9], [10], [11], [12], [13]. Thus, control of stem cell proliferation might be a common function of TRIM-NHL proteins and deregulation of muscle stem cell activity upon loss of TRIM32 could contribute to the formation of LGMD2H.

Stem cells of the adult muscle are termed satellite cells. These mononucleated cells are localized between the sarcolemma of the myofiber and the basal membrane that surrounds each muscle fiber [14]. Satellite cells display all the characteristics associated with stem cells, they are able to self-renew and to give rise to progeny that can undergo myogenic differentiation *in vitro* and *in vivo* [15], [16]. Following injury to skeletal muscle, quiescent satellite cells surrounding the damaged myofibers are activated and proliferate forming huge numbers of myoblast progeny cells which then differentiate and are either incorporated into existing damaged myofibers or undergo fusion with each other forming new myofibers [15]. A subset of these expanded progeny cells do not undergo differentiation and revert back to the quiescent state thus re-establishing the stem cell pool on the newly formed myofibers [17]. These characteristics enable satellite cells to regenerate damaged muscle as well as permitting growth.

A most recent study has suggested that the skeletal muscle pathology observed in *TRIM32* deficient mice could be a consequence of abnormalities in neuronal tissue [18]. However, the potential role for TRIM32 in directly regulating skeletal muscle stem cells has yet to be examined. Here, we demonstrated for the first time that TRIM32 expression is temporally regulated during satellite cell progeny expansion and is strongly induced during muscle differentiation *in vitro* and during muscular regeneration *in vivo*. We could show that TRIM32 is necessary and sufficient for myoblast differentiation in muscle progenitor cells which depends on the ubiquitination and degradation of its binding partner c-Myc. Finally, using two different lines of *TRIM32* null mice, we showed that loss of TRIM32 function causes a LGMD2H-like phenotype which is associated with dysfunctional muscle satellite cells. Moreover, skeletal muscle regeneration is greatly impaired in these mice, indicating an important role for TRIM32 in the regulation of skeletal muscle stem cells.

## Results

### TRIM32 is expressed in proliferating and differentiating myogenic cells

Previously, we have demonstrated that TRIM32 is necessary and sufficient for the differentiation of neural progenitor cells [7]. However, whether TRIM32 is also required for the differentiation of myogenic stem cells is largely unknown. As a first approach, we applied a well-established *in vitro* myofiber culture system to determine the dynamic expression of TRIM32 during the proliferation and differentiation of satellite cells.

**Figure 1:**
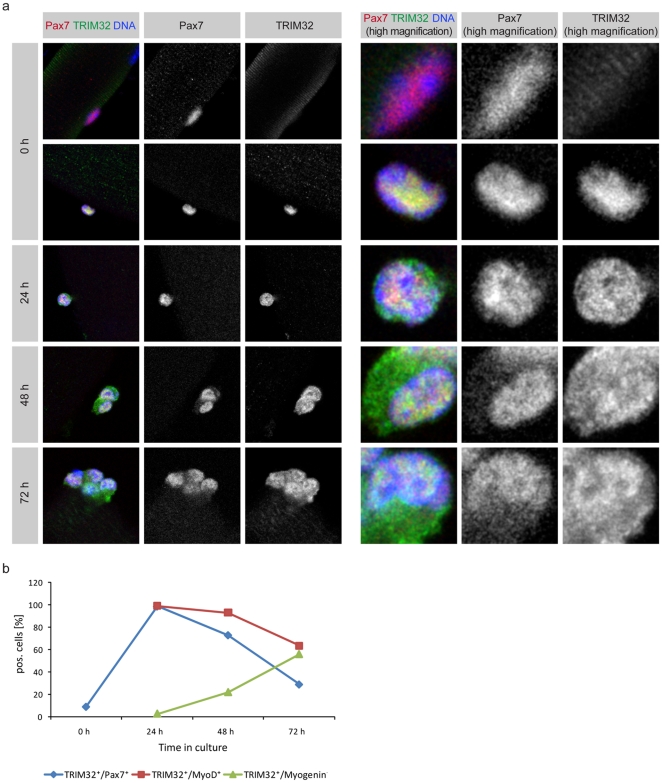
TRIM32 is expressed in satellite cells during regeneration *in vitro*. (a) Immunostainings of satellite cells on myofibers from wild type mice cultured for the indicated time (left grey boxes) and labelled with the indicated markers (upper grey boxes). The right panels show single nuclei in higher magnification. Note that here the anti-TRIM32 antibody 1137 (see methods) has been used. (b) Diagram showing the fraction of cells at each culture time point that were double-positive for TRIM32/Pax7, TRIM32/MyoD or TRIM32/Myogenin.

It has been shown previously, that TRM32 is expressed in mature muscle fibers [16]. In agreement with this observation we also saw a significant expression of TRIM32, showing a typical stripe-like patter, in isolated muscle fibers (Fig. S1). However, we were actually interested in the expression of TRIM32 in muscle progenitor cells. As in previous studies from ourselves and others, quiescent and non-committed satellite cells were marked using Pax7 immunoreactivity, proliferating cells were marked with Pax7 and MyoD and differentiating cells were labelled with Myogenin [17], [19], [16]. On freshly isolated mouse myofibers (at time point 0 h) harbouring quiescent satellite cells, around 90% of Pax7^+^ cells were negative for TRIM32, suggesting that TRIM32 is unlikely to be expressed in the quiescent satellite cell (Fig. 1a and b). However, 10% of satellite cells at this time point showed immunoreactivity for TRIM32 (Fig. 1a and b), which could reflect a small population of satellite cells that are activated upon muscle sample dissection (see discussion). Between 24 and 72 h of cultivation, satellite cells on isolated myofibers become activated, start to proliferate and produce clones of increasing size (Fig. 1a). Following 24 h of culture, when satellite cells are activated (100% of cells were positive for MyoD), 98.2% of satellite cells also showed double staining for Pax7 and TRIM32 (Fig. 1a and b). Interestingly these cells displayed a strong nuclear TRIM32 signal (Fig. 1a). After 48 and 72 h, high TRIM32 expression was also found in Myogenin^+^ cells (Fig. 1a, b and Fig. S1), demonstrating that TRIM32 is induced in activated satellite cells and also highly expressed in differentiating myoblasts. At 72 h, we also identified a group of cells (3.1%) that were Pax7^+^ but negative for TRIM32. These cells might have reverted back towards quiescence to replenish the pool of satellite cells, further indicating that TRIM32 is not expressed in quiescent satellite cells. At both the 48 and 72 h time points, all TRIM32 positive cells showed an additional strong cytoplasmic TRIM32 staining (Fig. 1a), suggesting a trafficking of TRIM32 during satellite cell-differentiation. However, the physiological relevance of this trafficking is currently unknown. To further prove the specificity of the described stainings, we used two other independent anti-TRIM32 antibodies that gave similar results (Fig. S1).

To address whether the above described expression pattern of TRIM32 in the myogenic lineage could be conserved between mice and primates, we analyzed the expression of TRIM32 in muscle fibers from the common marmoset *Callithrix jacchus*. Again, TRIM32 was only detected in the proliferating and differentiating satellite cells (Fig. S2).

Previous work has established that similar cellular mechanisms are involved in regulating both embryonic and foetal muscle growth and adult skeletal muscle regeneration [20], [19], [21]. In order to investigate whether TRIM32 is also involved in both stages of tissue development we analysed the expression of TRIM32 during primary (E14.5) and secondary (E18.5) myofiber formation, two key phases of murine skeletal muscle development in the embryo. We found that TRIM32 is expressed during mouse foetal skeletal muscle development in both differentiating progeny cells and newly formed myofibers identified through Myogenin and MHC type I co-staining, respectively (Fig. S3), suggesting the role for TRIM32 in adult skeletal muscle may be re-capitulated in the embryo.

### TRIM32 is strongly expressed during muscle regeneration

**Figure 2:**
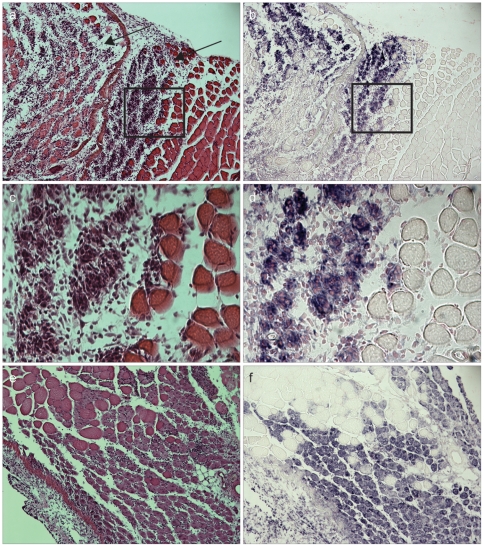
TRIM32 is induced during muscle regeneration *in vivo*. (a) and (c) Hematoxylin and Eosin (H&E) staining of regenerating TA muscle from wild type mice 3 days post cardiotoxin (CTX) injection. Arrows in (a) indicate the regions of CTX-induced muscle regeneration. (c) shows enlarged region of the boxed area in (a). (b) and (d) RNA in situ hybridization showing *TRIM32* expression (blue) in wild type mouse TA muscle 3 days post CTX injection. (d) shows enlarged regions of the boxed area in (b). (e) H&E staining of regenerating TA muscle from wild type mice 7 days post CTX injection. (f) RNA in situ hybridization showing *TRIM32* expression (blue) in wild type mouse TA muscle 7 days post CTX injection.

To determine whether TRIM32 expression is induced in proliferating satellite cells or myoblast cells *in vivo*, we analyzed the expression of TRIM32 during cardiotoxin (CTX)-induced muscle regeneration. Injection of CTX causes massive myofiber damage and subsequent necrosis followed by a period of extensive satellite cell proliferation and differentiation [15]. Following 3 and 7 days post injection, the regeneration response is at its highest as many infiltrating cells and proliferating myoblasts are apparent that surround newly forming skeletal muscle fibers (marked by fibers displaying centrally located nuclei) (Fig. 2). Using an RNA in situ hybridization approach, we observed markedly up-regulated *TRIM32* mRNA in areas of regenerating myofibers, but not in non-regenerating muscle tissue 3 days post injection (Fig. 2b and d). On day 7 post CTX injection, the newly forming myofibers with centrally located nuclei also showed strong staining for TRIM32 (Fig. 2f), suggesting that recently activated and fusing myogenic precursors express TRIM32 *in vivo*.

Taken together, this part of our work demonstrates that TRIM32 expression is highly induced during the activation of muscle satellite cells and is closely associated with muscle differentiation *in vivo*.

### Loss of TRIM32 function inhibits the differentiation of muscle satellite cells

To further investigate the role of TRIM32 in muscle satellite cells, we firstly applied a gene-targeting approach to create *TRIM32* null mutant mice in which the whole *TRIM32* coding sequence (Exon 2) was deleted (Fig. S4). A complete loss of TRIM32 expression in *TRIM32−/−* mice was confirmed by Western blot analysis (Fig. S4) and by Northern hybridization (data not shown). We then obtained single muscle fibers from these mutant mice to determine whether loss of TRIM32 function will affect the differentiation of muscle satellite cells. Additionally, we made use of *TRIM32* null mice that recently have been described [18].

**Figure 3:**
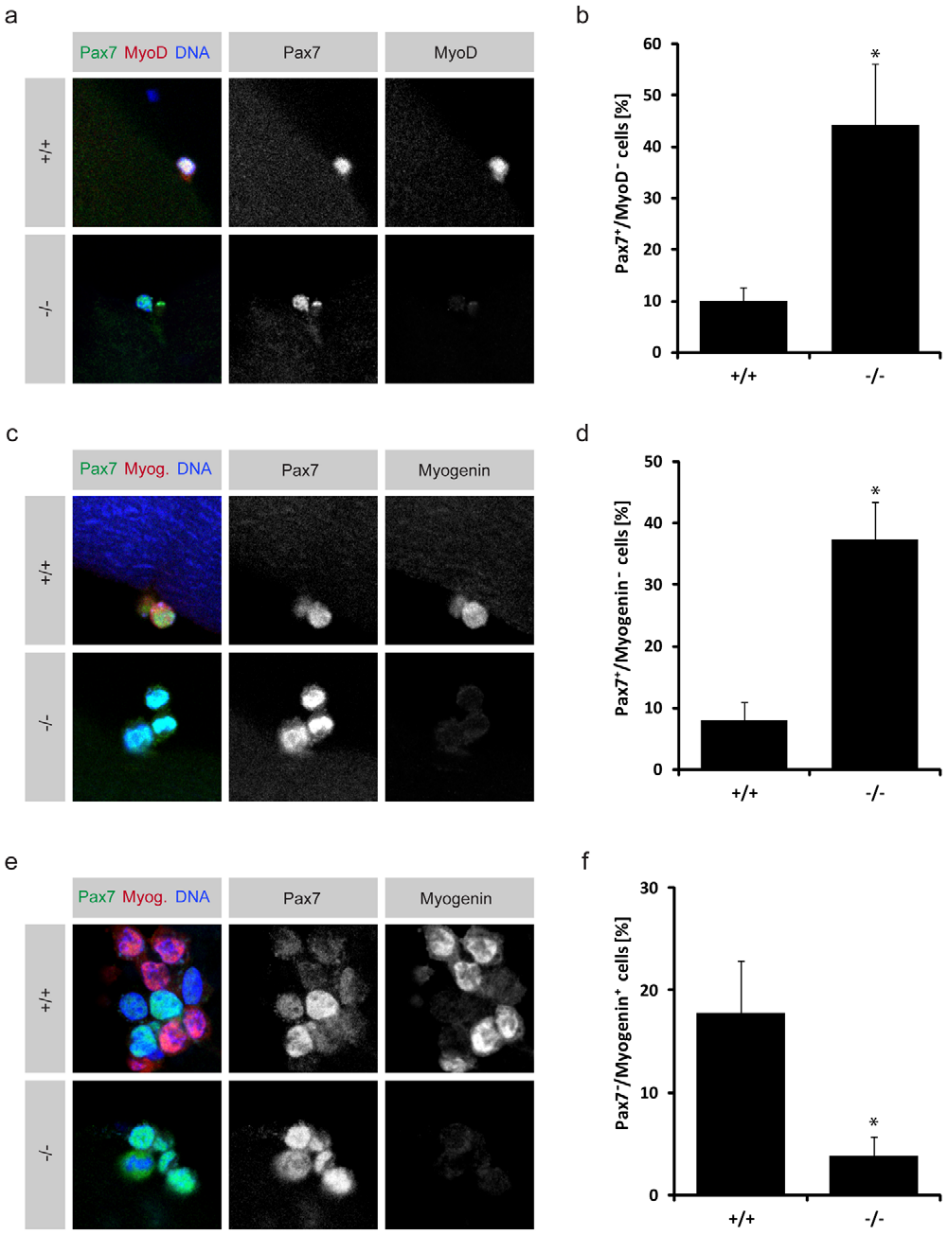
Differentiation is strongly reduced in satellite cell progeny on *TRIM32−/−* myofibers. (a), (c) and (e) Immunostainings of satellite cells on freshly isolated myofibers from wild type (+/+) and *TRIM32−/−* (−/−) mice cultured for 24 h (a), 48 h (c) and 72 h (e) and labelled with the indicated markers (upper grey boxes). (b), (d) and (f) Diagrams showing the relative frequency of Pax7^+^/MyoD^−^ cells on fibers cultured for 24 h (b), Pax7^+^/Myogenin^−^ cells on fibers cultured for 48 h (d) and Pax7^−^/Myogenin^+^ cells on fibers cultured for 72 h (f). In all cases cells on myofibers from wild type mice and *TRIM32−/−* mice are compared. (mean ± std; *P<0.001 compared to wild type).

On fibers from *TRIM32*+/+ mice at 24 h of cultivation, only 10.0% of the satellite cells (Pax7 positive) were still negative for MyoD, while all the others were co-stained with Pax7 and MyoD, indicating that the majority of *TRIM32*+/+ satellite cells underwent differentiation at 24 h of cultivation. In contrast, at the same time point on fibers from *TRIM32−/−* mice, 44.1% of the Pax7 positive satellite cells were still negative for MyoD (Fig. 3a and b). This result indicates that the *TRIM32* deficient satellite cells are delayed in initiating the myogenic differentiation program. This assumption is further supported by the observations from fibers that were cultivated for 48–72 h. At 48 h, only 8.0% of the satellite cells and their progeny were still negative for the muscle differentiation marker Myogenin on *TRIM32*+/+ myofibers, . In contrast, in the absence of TRIM32, 37.2% of the Pax7 positive cells were still negative for Myogenin (Fig. 3c and d). Consequently, at 72 h of cultivation already 17.8% of the progenitor cells on wild type fibers had downregulated Pax7 (they are Myogenin^+^/Pax7^−^), while only 3.9% of the *TRIM32* deficient progenitor cells were able to undergo this terminal differentiation at this time point (Fig. 3e and f). Additionally, a significant increase in apoptosis was observed in fiber cultures from TRIM32 deficient mice during myogenic differentiation (Fig. S6).

Therefore, our data strongly indicate that loss of TRIM32 function inhibits the differentiation and maturation of muscle satellite cells.

### TRIM32 induces differentiation in C2C12 muscle progenitor cells

**Figure 4:**
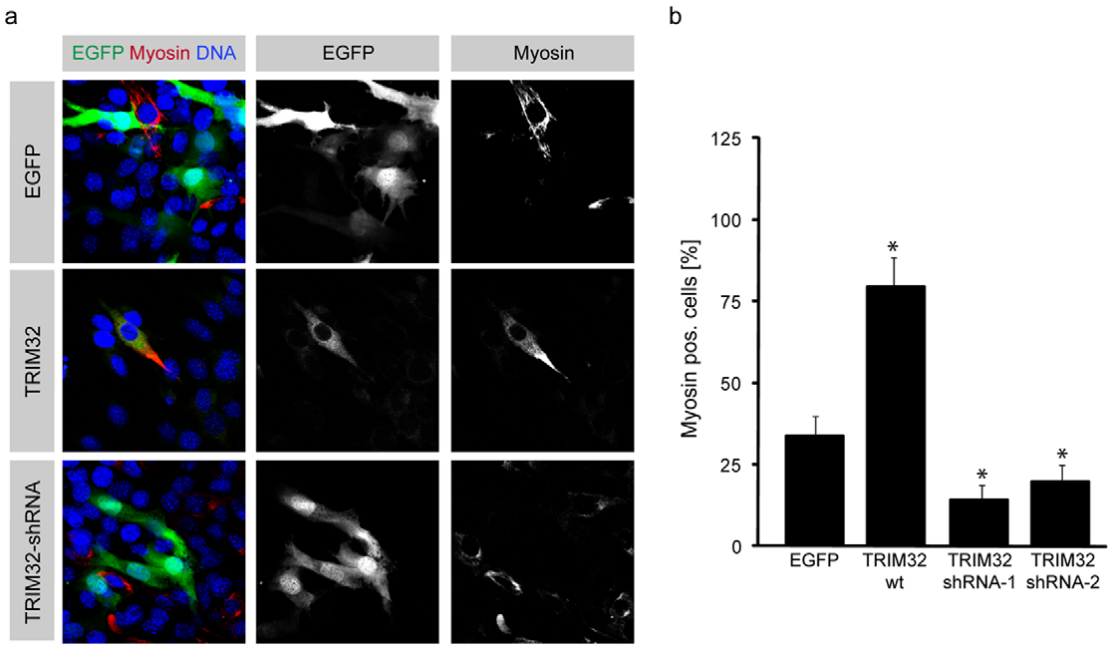
TRIM32 is necessary and sufficient to induce muscular differentiation. (a) Myoblast C2C12 cells were transfected with the indicated constructs (left grey boxes) and cultivated for three days in 2.5% HS. Immunostainings of the cells labelled with the indicated markers (upper grey boxes) are shown. (b) Diagram showing the fraction of transfected cells that undergo muscular differentiation (Myosin positive). (mean ± std; *P<0.001 compared to EGFP).

Since TRIM32 expression is significantly increased during the differentiation of C2C12 muscle progenitor cells (Fig. S5), we explored this well-characterized cell line to further characterize the role of TRIM32 in myogenic differentiation. It has been shown that the LGMD2H causing D489N mutation in TRIM32 [2] destabilizes the TRIM32 protein and leads to its degradation [5]. Consequently, a reduction of TRIM32 levels by an RNAi approach is suitable to model the disease situation. Therefore, we introduced TRIM32 overexpressing or RNAi constructs (both with an EGFP-tag) into C2C12 cells which were then differentiated for two days and stained for the differentiation marker Myosin heavy chain (MHC). In these assays, only transfected cells labeled by EGFP were analyzed. As shown in Figure 4, 33.7% of C2C12 cells transfected with a vector expressing only EGFP underwent muscle differentiation. However, upon transfection of TRIM32-EGFP, muscle differentiation was strongly increased, as 78.8% of the cells were positive for Myosin. Upon knock-down of TRIM32 by using two functional independent hairpin RNAi constructs against TRIM32, a strong inhibition of muscle differentiation was observed (shRNA #1: 14.2%; shRNA #2: 19.6%, as compared to 33% in control group) (Fig. 4a and b).

Taken together, our data demonstrate that TRIM32 is sufficient to induce the differentiation of C2C12 myoblast cells while upon knock-down of TRIM32 the differentiation of these cells is inhibited.

### c-Myc mediates TRIM32 induced muscle differentiation

**Figure 5:**
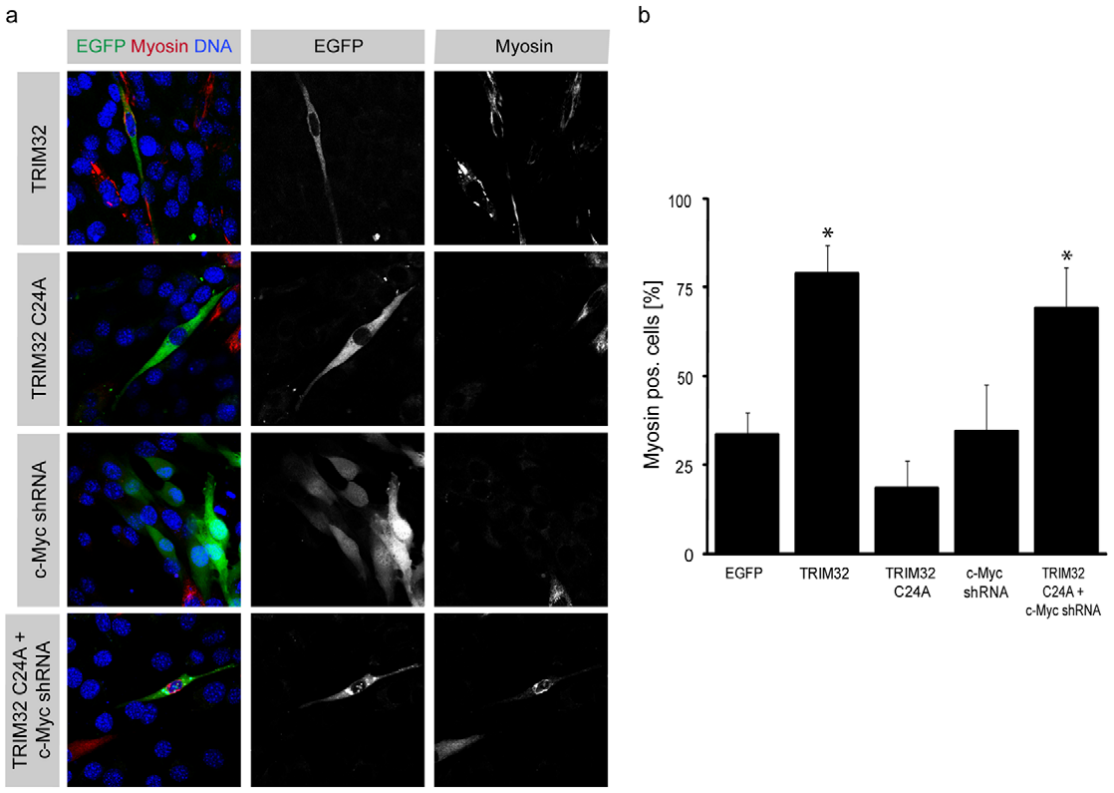
Degradation of c-Myc is critical for TRIM32 induced differentiation. (a) C2C12 cells were transfected with the indicated constructs (left grey boxes) and cultivated for three days in 2.5% HS. Immunostainings of the cells labelled with the indicated markers (upper grey boxes) are shown. (b) Diagram showing the fraction of transfected cells that undergo muscular differentiation (Myosin positive). (mean ± std; *P<0.001 compared to EGFP).

We then sought to demonstrate how TRIM32 is involved in the differentiation of myoblasts by using the C2C12 cell culture system. Previously we have shown that TRIM32 can ubiquitinate c-Myc, targeting the protein for breakdown and that this degradation of c-Myc is critical for differentiation in neural progenitor cells [7]. Furthermore, c-Myc has been demonstrated to block the action of MyoD- and Myogenin-mediated skeletal muscle stem cell differentiation [22]. Therefore, we firstly asked whether TRIM32 is also able to mediate poly-ubiquitination of c-Myc in C2C12 cells. Indeed, when TRIM32 and c-Myc are coexpressed in C2C12 a strong poly-ubiquitination of c-Myc is detectable (Fig. S7). In contrast this poly-ubiquitination is no longer detectable when the TRIM32 C24A mutant that has no ubiquitin ligase activity [7] is expressed. Furthermore, poly-ubqiutination of c-Myc is detectable during differentiation of C2C12 cells (Fig. S7b). Therefore, we conclude that TRIM32 is directly poly-ubiquitinating c-Myc in muscle progenitor cells. Next we asked whether the regulation of c-Myc by TRIM32 could be required for the differentiation of C2C12 myoblast cells. To this end, we overexpressed TRIM32 or TRIM32 C24A in C2C12 cells and differentiated these cells for 2 days. In control cells, expressing only EGFP 33.7% of the EGFP transfected cells were positive for the differentiation marker Myosin. In contrast expression of wild-type TRIM32 induces differentiation in 78.8% of the cells (Fig. 5a and b). To characterize whether ubiquitination of c-Myc by TRIM32 is indeed involved in the TRIM32-induced muscle differentiation, we knocked down c-Myc with RNAi constructs and combined this knock-down with the overexpression of the ubiquitin ligase defective TRIM32 C24A mutant). Overexpression of TRIM32 C24A alone did not increase muscular differentiation (only 18.4% of the C2C12 cells expressed Myosin). However, when the TRIM32 C24A expressing vector was co-transfected with knock-down constructs for c-Myc in C2C12 cells, 69.3% of transfected cells differentiated (Fig. 5a and b). Interestingly, knock-down of c-Myc alone was not sufficient to induce muscular differentiation. These results support the model that TRIM32 induces differentiation by marking c-Myc for degradation via the ubiquitin-proteasome-system. However, they also clearly show that TRIM32 has a second, ubiquitin-ligase independent function that is important to support differentiation (see discussion).

### *TRIM32* deficient mice develop a LGMD2H-like myopathy

Thus far we have demonstrated that TRIM32 is expressed in proliferating and differentiating skeletal muscle stem cells and is necessary for their normal lineage progression into mature MHC-expressing myofibers. Moreover we provide evidence that TRIM32 induces the differentiation of skeletal muscle progenitor cells through the degradation of c-Myc via the ubiquitination pathway. Recently, it has been shown that *TRIM32* deficient mice harbour a phenotype similar to that observed in LGMD2H [18]. However, it is unknown whether this phenotype could be brought about through abnormal skeletal muscle stem cell behaviour.

To address this, we firstly performed extensive histological analyses on our established *TRIM32* null mice to determine whether these mutant mice develop a similar myopathy as seen in human LGMD2H patients.

**Figure 6:**
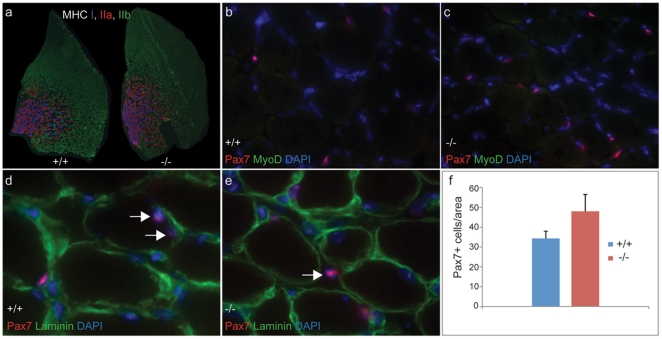
Loss of TRIM32 function causes an increase in muscle satellite cells and their mislocalization. (a) Immunostainings with antibodies against different MHC isoforms shows no changes in TA muscle morphology and fiber composition between one month-old wild type (+/+) and *TRIM32* deficient mice (−/−). (b–e) Immunostainings of muscle satellite cells in TA muscle sections from one month-old wild type (+/+) and *TRIM32−/−* (−/−) mice labelled for the indicated markers. Arrows in (d) indicate two satellite cells located beneath the basal lamina and the arrow in (e) indicates one satellite cell located outside the basal lamina.

We then analyzed the status of muscle satellite cells in *TRIM32−/−* mice at different postnatal developmental stages. We found that one-month-old *TRIM32−/−* mice had significantly increased numbers of Pax7^+^ satellite cells as compared to wild type controls (Fig. 6b, c and f). These cells were MyoD negative, indicating they were not induced by muscle regeneration. Therefore we conclude that a dysregulated satellite cell proliferation occurred in the absence of TRIM32. Interestingly, many of these satellite cells were found to be located outside the basal lamina (normally satellite cells are all beneath the basal lamina) (Fig. 6d and e), In 2–3 month-old *TRIM32* mutant mice, we did not find the increased number of Pax7^+^ satellite cells, but we still observed numerous satellite cells mis-located in the interstitial space (data not shown). These data clearly demonstrate that loss of TRIM32 function causes abnormal muscle stem cell behavior *in vivo*.

### *TRIM32* deficient mice show delayed skeletal muscle regeneration

**Figure 7:**
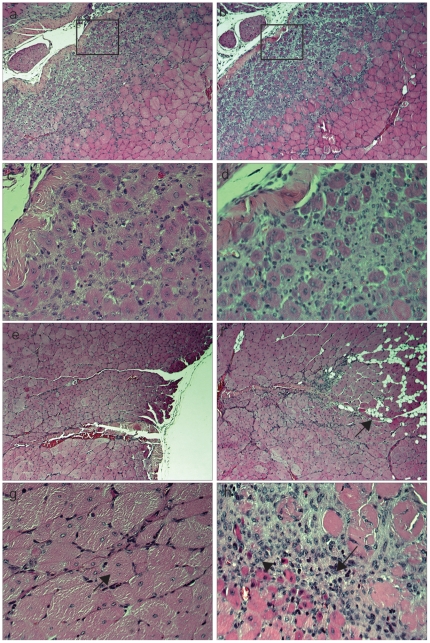
*TRIM32−/−* mice show delayed and impaired skeletal muscle regeneration. (a)–(d) H&E staining of transverse cross sections through CTX-injured TA muscles 5 days post injection from wild type (a) and (c) and *TRIM32−/−* (b) and (d) mice. (c) and (d) show enlarged regions of the boxed areas in (a) and (b), respectively. (e)–(h). H&E staining of transverse cross sections through CTX-injured TA muscles 16 days post injection from wild type (e) and (g) and *TRIM32−/−* (f) and (h) mice. (g) and (h) show enlarged regions of (e) and (f), respectively. Arrow in (g) indicates centrally nucleated myofiber characteristic of efficient muscle regeneration in wild type TA. In contrast, some regions of injured *TRIM32−/−* TA display small centrally nucleated myofibers (h, arrowhead), adipocyte-like cells (f, arrow) or fibrosis (h, arrow).

To further determine whether the satellite cells in *TRIM32−/−* mice are capable of providing myoblasts which will undergo normal muscle regeneration, we carried out a similar set of experiments using the CTX-induced muscle injury model and compared the regenerative response in both wild type and *TRIM32−/−* TA muscles respectively. Strikingly, we observed a delay in the regeneration response in the absence of TRIM32, whereby TA muscles 5 days post-injection of CTX displayed smaller newly formed myofibers and showed larger regions of tissue containing infiltrating cells (compare Fig. 7a to b and c to d). Moreover, 16 days post injection, when wild type control skeletal muscle has fully regenerated and only harbors few centrally located myonuclei, *TRIM32−/−* muscle continued to display regions of non-regenerated myofibers that contained many single cells with the characteristics of cellular infiltrate (Fig. 7e–h). Finally, when we looked at 16-days post injection, we observed the formation of adipocyte-like cells within the muscle tissue of *TRIM32−/−* animals. These cells were never seen in wild type muscle (Fig. 7e and f). Similar results were obtained after CTX injection in gastrocnemius muscles (unpublished data). Taken together, these data show that TRIM32 is essential for the normal progression of skeletal muscle regeneration *in vivo* and that loss of TRIM32 function, by perturbing the differentiation of myoblast cells into muscle, may lead to the conversion of these cells into other lineages.

## Discussion

In this work, we investigated the function of TRIM32, a gene that is often mutated in patients with LGMD2H, during skeletal muscle regeneration. Our data has revealed a number of novel findings which extend our understanding of skeletal muscle stem cell biology and provide an explanation for the LGMD2H phenotype. Firstly, we have shown that TRIM32 is upregulated in muscle stem cells (satellite cells) during their proliferation and differentiation, and this upregulation was also observed in regenerating muscles in which muscle satellite cells are actively involved. Interestingly, this dynamic expression pattern is also conserved in the common marmoset *Callithrix jacchus*. Using different approaches, we have further demonstrated that TRIM32 is involved in muscle differentiation and that this differentiation probably depends on the ubiquitination and degradation of c-Myc. Finally, we provided evidence that *TRIM32* knock-out mice developed a myopathy similar to that presented by LGMD2H patients and that muscle regeneration in these mice was greatly delayed and impaired. Based on these experimental findings, we propose that loss of TRIM32 function in the muscle stem cells will impair their functionality. This dysfunction of the muscle stem cells could be the main cause for the aetiology of LGMD2H. Throughout normal adult life, small lesions in the muscle frequently occur and are repaired by satellite cells. However, in *TRIM32* deficient mice or in LGMD2H patients, satellite cells and their progeny are not able to properly differentiate into mature muscle cells and therefore lesions are not repaired. As a consequence, the status of the muscle becomes progressively worse over time until it becomes apparent as a dystrophy.

In this study, we present a temporal and spatial expression analysis for TRIM32 during the lineage progression of satellite cells. In 90% of quiescent satellite cells we observed no TRIM32 expression, which strongly indicated that TRIM32 is not expressed in this specific cell population. However, we do find that around 10% of these cells express TRIM32. Two possible explanations for this are apparent. Either TRIM32 is expressed in a subset of resting satellite cells (an unlikely explanation as we failed to find TRIM32 expression on resting muscle transverse sections) or TRIM32 is initiated very early in the regenerative response to injury. This second explanation is more likely as myofibers take at least 2 h to be isolated and fixed, enough time to initiate the expression of a protein as previously observed in the case of MyoD, that is initiated just 2 h following myofiber culture [19]. By 48 h post-myofiber isolation, when most progeny cells are proliferating [17], all progeny express TRIM32. Moreover, low levels of TRIM32 were observed in proliferating C2C12 cells, suggesting that TRIM32 is not inhibitory to proliferation at lower levels. However, as myoblasts differentiate TRIM32 is upregulated, as we have observed in C2C12 cells. Interestingly, we observed four distinct populations of myogenic cells following 72 h of single myofiber culture. The largest subset of TRIM32^+^ cells at this time point co-expressed the differentiation marker Myogenin, however Pax7^+^/Myogenin^−^ cells also showed TRIM32 expression at this stage. Interestingly, a small subset of non-differentiated Pax7^+^ cells were noted that were also negative for (and therefore had switched off) TRIM32 expression. These cells showed the characteristics of quiescent muscle stem cells as observed on resting myofibers. It is reasonable to propose that this subset of cells corresponds to the self-renewing population that goes on to replenish the satellite cell pool on the myofiber [16] and that the down-regulation of TRIM32 occurs prior to these cells taking up their quiescent state.

These observations fit well with the established model for satellite cell lineage progression that is regulated through the balance of MyoD and Pax7 protein expression within the satellite cell [23] [24]. A similar ratio of Pax7∶MyoD within the myoblast is necessary for satellite cell proliferation, a situation that we observe in the presence of low levels of TRIM32. However, as TRIM32 expression increases within the majority of myoblasts, it acts to ubiquitinate c-Myc causing its breakdown and thus the c-Myc inhibition of MyoD expression [22] is lost. As a consequence, MyoD expression levels increase within the cell and the ratio of Pax7∶MyoD is reduced, thus inhibiting proliferation and inducing differentiation through Myogenin expression. In the absence of TRIM32, this balance of Pax7∶MyoD is dysregulated, because degradation of c-Myc is not initiated, MyoD levels do not increase and Myogenin expression is not activated. Consequently, differentiation of the satellite cells is impaired or delayed. Interestingly, we also found that TRIM32 is expressed during foetal skeletal muscle development in both differentiating progeny cells and newly formed myofibers (Fig. S3), suggesting the role for TRIM32 in adult skeletal muscle may be re-capitulated in the embryo. Nevertheless, compensatory proteins must enable the development of skeletal muscle in the absence of TRIM32.

We have shown that TRIM32 is expressed in both proliferating and differentiating satellite cell progeny. Since in the absence of TRIM32, Pax7^+^ muscle stem cells fail to differentiate (Fig. 3), one may hypothesise that this lack of differentiation could lead to tumour formation. However, we never observed tumours in the muscle or any other tissue in *TRIM32* knock-out mice. The reason for this initially surprising observation is probably that once satellite cells become activated, they are unable to develop further into a more differentiated state and instead undergo apoptosis following the loss of TRIM32 expression. Such a vertebrate-specific protection mechanism to prevent tumourigenesis in response to stem cell over-proliferation has been suggested previously [25]. However, the underlying molecular mechanism is still not clear.

We show that despite *TRIM32−/−* satellite cells being delayed in their differentiation program, they nevertheless eventually form some differentiated cell progeny. These results would seem to be contradictory. However, due to the intimate link between proliferation and differentiation, particularly in the myogenic lineage, we suggest the following possible explanations. Firstly, in order to form large numbers of differentiating progeny, the expansion of the satellite cell population must take place.

We have observed that in the absence of TRIM32 initial lack of differentiation does occur and that the affected satellite cells fail to initiate appropriate Myogenin expression. It is well established that satellite cells, when given the correct cues, are able to differentiate into other cell types including adipocytes [26], therefore we cannot rule out the possibility that lineage conversion is taking place in a subset of *TRIM32−/−* satellite cells. Secondly, we have also observed the necessity for MyoD, a member of the myogenic regulatory factor family of proteins essential for normal rates of myoblast differentiation, in the proliferation of the myoblast pool. Therefore, if TRIM32 is acting to induce differentiation through the indirect upregulation of MyoD and subsequently Myogenin, its absence may perturb levels of differentiation through preventing MyoD levels to build up in the cell. It is clear that Myogenin expression does occur (albeit at a lower frequency) in the absence of TRIM32. Therefore other factors may compensate for the loss of TRIM32 and enable this process to occur, even though these factors do not enable normal numbers of differentiated progeny to develop.

We showed that TRIM32-induced differentiation involves the poly-ubiquitination of c-Myc within muscle progenitor cells. The knock-down of c-Myc alone is not sufficient to induce differentiation. Therefore one could speculate that additionally to c-Myc, other proteins have to be ubiquitinated by TRIM32. However, because the overexpression of a mutant of TRIM32 that is no longer able to ubiquitinate, together with a knock-down of c-Myc was sufficient to induce differentiation, this possibility seems to be unlikely. Together these data demonstrate that a TRIM32 mediated reduction in the levels of c-Myc (via poly-ubiquitination) is necessary but not sufficient for induction of differentiation. Previously we have shown that TRIM32 also binds to Argonaute-1 and that this binding leads to the activation of the micro-RNA Let-7a [7]. Therefore it is possible that activation of micro-RNAs by TRIM32 is also critical for muscular differentiation. However unlike in neural progenitor cells, in C2C12 cells the expression of Let-7a is not sufficient to induce muscular differentiation (data not shown). Nevertheless, this does not rule out the possibility that activation of micro-RNAs other than Let-7a are necessary for myoblast differentiation. Finally, we demonstrate that in the absence of TRIM32, myogenic regeneration is greatly perturbed highlighting the importance of TRIM32 specifically in this process. Moreover, key phenotypic features of *TRIM32−/−* regenerating muscle correlate well with our *in vitro* data. Firstly, regeneration is delayed and it takes longer for *TRIM32−/−* myofibers to reach the same size as their wild type equivalents. Therefore, the number of cells undergoing myogenic differentiation must be reduced *in vivo*. Secondly, regions of skeletal muscle progenitors and/or cellular infiltrate persist in the tissue in the absence of TRIM32 (noted at 16 days post injury – Fig. 7), far beyond the time when wild type regeneration has been completed (approximately 10 days) suggesting that these cells have failed to differentiate even by this late stage. Finally, we noted the presence of regions containing adipocyte-like cells within the skeletal muscle tissue at 16 days post injury in the absence of TRIM32. These data suggest that when the differentiation potential of myoblasts is perturbed through loss of TRIM32, these residual pockets of myoblasts may take up a separate adipogenic lineage. The fact that these cells become adipocytes and not myofibroblasts, connective tissue or endothelial tissue is not surprising, as recently links between the myogenic and adipogenic lineages have been realised with brown fat and skeletal muscle sharing common ancestry [27]. These data clearly demonstrate that TRIM32 is necessary for satellite cell-mediated skeletal muscle regeneration and suggest that the neuronal phenotype observed in the *TRIM32−/−* mouse [18] is not the only causative reason for the apparent myopathy.

To summarise, this study has demonstrated that the ubiquitin ligase TRIM32 plays a key role in lineage progression of satellite cells and is essential for their differentiation thus expanding our knowledge about how satellite cell-mediated skeletal muscle regeneration is regulated. Moreover, we provide an explanation for the phenotype observed in LGMD2H, thus greatly enhancing our understanding of this pathology and therefore providing new avenues of research into potential therapies of this disease.

## Materials and Methods

### Plasmids

The following plasmids were used: pEGFP-N1 (Clontech), pEGFP-TRIM32 [7], pEGFP-TRIM32 C24A [7], pEGFP-TRIM32 D489N (generated by side directed mutagenesis from wildtype TRIM32) pRetroSuper c-Myc shRNA (Martin Eilers), pcDNA3 c-Myc (Martin Eilers), pRNAT-H1.4 TRIM32-shRNA #1 (GenScript) and pRNAT-H1.4 TRIM32-shRNA #2 (GenScript).

### Generation of Mice Deficient for *TRIM32*

The mouse *TRIM32* genomic fragments were PCR-amplified from the genomic DNA of R1 ES cells (on 129S1 background) with a high fidelity polymerase from Clontech to build a gene-targeting vector, which was designed to replace a 5 kb genomic fragment containing exon 2 of *TRIM32* with an *SA-IRES-βgeopA* expression cassette. Southern blot analysis was carried out to screen for the presence of a disrupted *TRIM32* locus using *Bam*H1/*Kpn*1 (for the 5′ external probe) and *Nsi*1 digestion (for the 3′ external probe). We used two independently targeted ES cell clones to generate chimeric mice that subsequently were bred with 129S1 females to obtain germ-line transmission. The phenotypes of *TRIM32−/−* mutants derived from both targeted ES cell lines were indistinguishable. All the shown experiments have been conducted with those mice. All mouse experiments were performed in accordance with procedures approved by the University of Manitoba Animal Care and Use Committee.

Additionally, *TRIM32*-null mice that have been described recently [18] were used to repeat and verify the results shown in Figures 3, S1 and S6.

### Cardiotoxin (CTX)-induced muscle injury and regeneration

25 µl CTX (10 µM; Latoxan) were injected into the tibialis anterior (TA) or the gastrocnemius muscles of mice. Mice were sacrificed and the injected muscle tissues were collected at the indicated time points,fixed with formalin overnight and embedded in paraffin. Sections were stained with hematoxylin-eosin.

### Myofiber culture (mice)

The isolation and culture of myofibers has been described previously [19]. Briefly, EDL muscles were dissected and placed in 0.1% collagenase type I (Sigma) in DMEM and incubated for approximately 1.5 h at 37°C. Using graded glass pipettes, myofibers were liberated from the muscle bulk, washed in DMEM and either fixed in 4% Paraformaldehyde (PFA) in PBS for 15 min or cultured. For myofiber culture, single isolated myofibers were placed in floating culture in 24 well plates with 2–4 fibers per well in 0.5 ml single fiber culture medium (SFCM - DMEM containing GLUTAMAX, 100 µg/ml penicillin streptomycin, 10% (v/v) horse serum and 0.5% (v/v) chick embryo extract) at 37°C, 5% CO_2_ for either 24, 48 or 72 h. Myofibers were fixed in 4% PFA/PBS for 15 min and processed for immunocytochemistry.

### Myofiber culture (*Callithrix jacchus*)

Marmoset monkeys (*Callithrix jacchus*) were raised in the institutional breeding facility of the Centre of Reproductive Medicine and Andrology. Breeding (Prof. Dr. Stefan Schlatt), maintenance and experimental procedures were performed in accordance with the German Federal law on the Care and Use of laboratory animals. A license for the marmoset breeding colony was obtained from local authorities (Veterinär- und Lebensmittelüberwachungsamt der Stadt Münster). The license to sacrifice marmosets as tissue donors for scientific experiments was granted by the Landesamt für Natur, Umwelt und Verbraucherschutz Nordrhein-Westfalen (8.87–50.10.46.09.018). The animals were deeply sedated using ketamine and killed by exsanguination. Muscle tissue from adult marmosets was obtained from local colonies at the Institute of Reproductive Medicine, University of Münster. Cultures of myofibers from this tissue were obtained and treated as described above for the mouse tissue.

### Immunohistochemistry and RNA in situ hybridization

Fixed myofibers or muscle tissue cryosections were permeabilised in a solution of 20 mM Hepes, 300 mM sucrose, 50 mM NaCl, 3 mM MgCl_2_ and 0.5% Triton-X100 (pH 7) at 4°C for 15 min. Fibers were washed in PBS and non-specific binding was blocked using wash buffer (5% (v/v) Foetal Calf Serum (FCS)/PBS with 0.05% (v/v) Triton-X100) for 30 min. The following primary antibodies were used: anti-Pax7 (Neuromics, 1∶100), polyclonal rabbit anti-MyoD (Santa Cruz Biotechnology-M-318, 1∶200), polyclonal rabbit anti-Myogenin (Santa Cruz Biotechnology-M-225, 1∶200) and anti-Myosin Heavy Chain (A4.1025, developmental studies hybridoma bank 1∶4). Alexa-fluorophore conjugated antibodies (Invitrogen) and HRP-coupled secondary antibodies (MoBiTec) were used for visualisation. The rabbit anti-TRIM32 antibody (1137, directed against the N-Terminus of the protein) was described previously [7]. This antibody was used to detect TRIM32 when nothing else is indicated. Additionally, we used a commercially available anti-TRIM32 antibody (M09, Abnova; Fig. S1b) and a self-made anti-TRIM32 antibody (3150; Fig. S1c) directed against the C-terminal part of the protein (peptide antigen: VKIYSYHLRRYSTP).

All antibodies were diluted in wash buffer and samples were incubated in primary antibodies for 18 h at 4°C. The anti-rabbit secondary antibody was incubated for 1 h at room temperature and all fluorescent antibody labeling was carried out at room temperature for 45 minutes. Myofibers and tissue sections were mounted using fluorescent mounting medium (DakoCytomation). Nuclear visualization was carried out through either DAPI (2.5 µg/ml) or Hoechst 33258 (Invitrogen) DNA stains.

RNA in situ analysis of frozen sections of mouse muscles were performed according to established protocols [28], with sense and antisense digoxigenin-labeled riboprobes which were *in vitro* transcribed from the full-length mouse *TRIM32* coding sequence.

### C2C12 cell cultures

C2C12 cells were grown in DMEM supplemented either with 15% heat-inactivated FCS (proliferation) or 2.5% heat-inactivated HS (differentiation), 2 mM L-glutamine, 100 U/ml penicillin and 100 µg/ml streptomycin. The cells were transfected with Fugene6 (Roche) according to the instructions of the manufacturer. C2C12 cells were processed for immunohistochemistry by fixation with 4% PFA in 120 mM phosphate buffer, pH 7.4 (PBS), permeabilized with 0.05% Triton X-100 in PBS, blocked with 10% fetal calf serum in PBS and subjected to immunohistochemistry stainings with primary antibodies diluted in blocking solution. Images were collected by confocal microscopy using LSM software (Zeiss); image analysis was performed with the LSM software, Adobe Photoshop and the IMAGE J software. For Western Blotting C2C12 cells were lysed after two days of cultivation under proliferation or differentiation conditions with lysis buffer 1 (2% Triton X-100 and Complete protease inhibitor cocktail (Roche) in PBS) for 30 min at 4°C. For qPCR analysis C2C12 cells were lysed after two days of cultivation under proliferation or differentiation conditions. The total mRNA was isolated with the miRNeasy Kit (Quiagen) and qPCR was performed with commercially available probes (Sigma) specific for TRIM32 or GAPDH (control).

### Immunoprecipitation and Western Blot

For the ubiquitination assay, C2C12 cells were transfected using Turbofect (Fermentas) according to the manufacturer's instructions and were lysed 48 h after transfection with nuclear lysis buffer containing 50 mM TRIS (pH 7.5), 0.5 M NaCl, 1% NP-40, 1% DOC, 0.1% SDS, 2 mM EDTA and complete protease inhibitor (Roche) in H_2_O for 30 min at 4°C. After lysis of the cells, the percentage of SDS was reduced to 0.07% by adding TRIS/EDTA. For immunoprecipitation, the cell lysates were incubated with the precipitation antibody for 4 h at 4°C and overnight with protein-G Agarose beads (GE Healthcare). Afterwards, bound proteins were eluted by boiling in protein sample buffer.

For immunoprecipitation assays from differentiated cells, C2C12 cells were differentiated 24 h after transfection using DMEM supplemented with 2.5% heat-inactivated HS, 2 mM L-glutamine, 100 U/ml penicillin and 100 µg/ml streptomycin. 24 h after differentiation, the cells were incubated with a final concentration of 10 µM of the proteasome inhibitor MG-132 (Sigma) for 7 h before lysis.

To ensure loading of equal amounts of protein for SDS-PAGE and Western Blotting the total concentration of proteins was determined with a BCA protein assay kit (Thermo Scientific) according to the manufacturer's instructions. Furthermore, equal amounts of blotted protein were verified by Ponceau S (Sigma) staining on the membrane. Analysis of Western Blots was done using Image J software.

## Supporting Information

Figure S1 **Coexpression of TRIM32 and MyoD or Myogenin and absence of TRIM32 in myofibers of** ***TRIM32−/−*** **mice.** (a) Immunostainings of myofibers from wild type mice cultured for 0 h and labelled with the indicated markers (upper grey boxes). Note that here the anti-TRIM32-1137 antibody has been used. (b) Immunostainings of satellite cells on myofibers from wild type mice cultured for the indicated time (left grey boxes) and labelled with the indicated markers (upper grey boxes). Note that here the anti-TRIM32 antibody M09 has been used. (c) Immunostainings of satellite cells on myofibers from wild type (+/+) and *TRIM32−/−* (−/−) mice labelled with the indicated markers (upper grey boxes). Note that here the anti-TRIM32 antibody 3150 has been used.
(TIF)

Figure S2 **The expression pattern of TRIM32 is conserved in the Common Marmoset** ***Callithrix jacchus*** **.** Immunostainings of satellite cells on Marmoset myofibers cultured for 24 h and labelled with the indicated markers (upper grey boxes).
(TIF)

Figure S3 **Expression of TRIM32 during muscle development.** (a) Immunostainings of cross sections from wild type E14.5 Lower Hindlimb and E18.5 EDL Muscle labelled with the indicated markers (upper grey boxes). (b) Immunostainings of cross sections from wild type E18.5 EDL Muscle labelled with the indicated markers (upper grey boxes).
(TIF)

Figure S4 **Gene-targeting strategy for creating the** ***TRIM32*** **null allele.** (a) Schematic representation of the *TRIM32* genomic locus, the gene-targeting vector and the mutated locus with Exon 2 replaced by the *SA-IRESβgeo* cassette. (b) Southern Blot analysis of ES clones with insertion of targeting vector. Both 5′ and 3′ probes gave predicted digestion patterns. (c) Western Blot analysis of TRIM32 expression in cell lysates from mouse embryonic fibroblast derived from *TRIM32* knock-out mice, demonstrating complete loss of TRIM32 expression in *TRIM32−/−* cells.
(TIF)

Figure S5 **Expression of TRIM32 is upregulated during differentiation of C2C12 cells.** (a) Immunostainings of C2C12 cells cultivated for 0 days, two days or 5 days under differentiation conditions and labelled with the indicated markers (upper grey boxes). (b) Western Blot analysis of TRIM32 expression in C2C12 cells cultivated for 0 days, two days or 5 days under differentiation conditions. (c) Diagram showing the relative levels of TRIM32 protein in C2C12 cells (Western Blot measurement as in (c)) cultivated under differentiation conditions. Ponceau S staining was used to normalize the protein levels.
(TIF)

Figure S6 **Increased apoptosis in satellite cells of** ***TRIM32−/−*** **mice.** (a) Immunostainings of satellite cells on myofibers from wild type (+/+) and *TRIM32−/−* (−/−) mice cultured for 24 h and labelled with the indicated markers (upper grey boxes). (b) Diagram showing the fraction of satellite cells on myofibers of wild type (+/+) and *TRIM32−/−* (−/−) mice undergoing apoptosis (cleaved Caspase 3 positive) at 24 h, 48 h and 72 h.(mean ± std; *P<0.001 compared to wild type).
(TIF)

Figure S7 **TRIM32 binds to and ubiquitinates c-Myc in C2C12 cells.** (a) Western blot analysis of C2C12 cells transfected with the indicated constructs and immunoprecipitation of c-Myc with an anti-c-Myc antibody. Antibodies against c-Myc and HA were used for detection of ubiquitinated c-Myc after immunoprecipitation and an antibody against EGFP was used to detect the different TRIM32 constructs (arrow) that bound to c-Myc. In the lysate, the different TRIM32 constructs (arrow) and EGFP were detected with an anti-EGFP-antibody. Unspecific bands are indicated by asterisks. The GAPDH western blot is shown as loading control. (b) Western blot analysis of C2C12 cells transfected with HA-tagged Ubiquitin and cultivated for two days under differentiation conditions. The cells were treated for seven hours with the proteasome inhibitor MG-132 and c-Myc was immunoprecipitated using an anti-c-Myc antibody. Antibodies against c-Myc and mono- and poly-ubiquitinated proteins were used for detection of ubiquitinated c-Myc after immunoprecipitation.
(TIF)
