# Supplementary figures and images for: TRIM32 Regulates Skeletal Muscle Stem Cell Differentiation and Is Necessary for Normal Adult Muscle Regeneration
Source: PLoS One. 2012 Jan 27;7(1):e30445. doi: 10.1371/journal.pone.0030445 (PMC3267731; doi:10.1371/journal.pone.0030445)

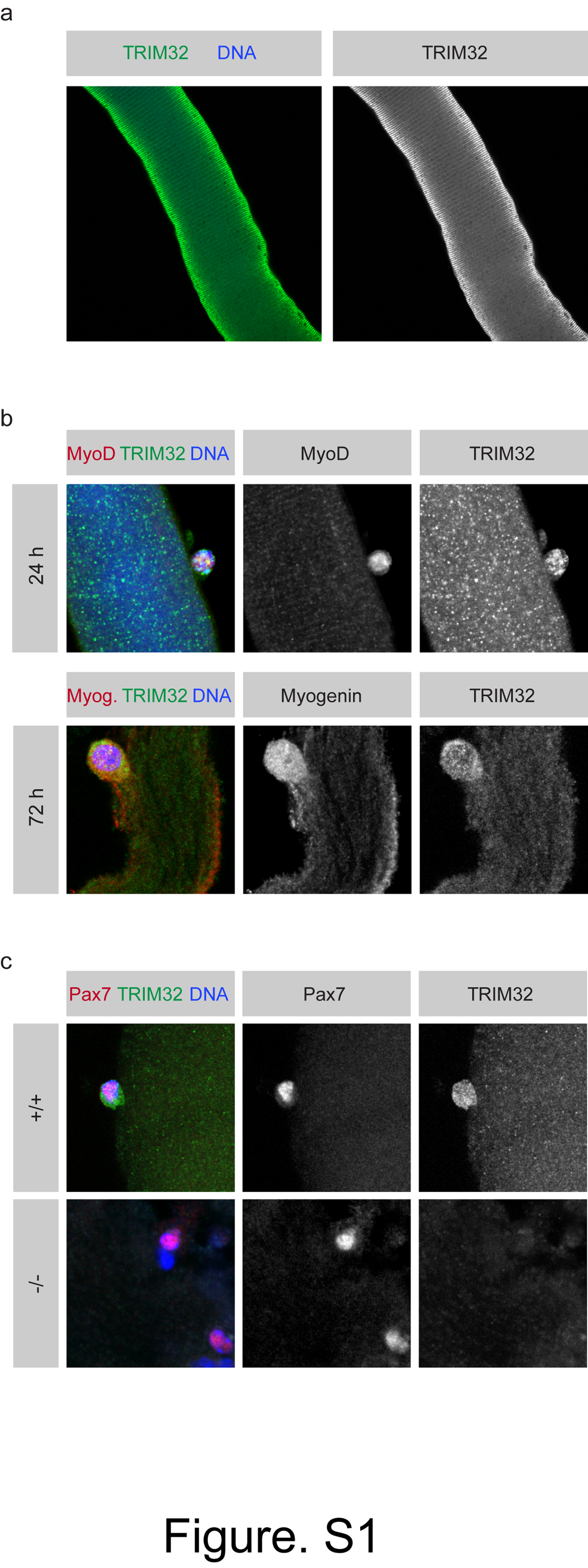

Supplement: Figure S1 — Coexpression of TRIM32 and MyoD or Myogenin and absence of TRIM32 in myofibers of TRIM32−/− mice. (a) Immunostainings of myofibers from wild type mice cultured for 0 h and labelled with the indicated markers (upper grey boxes). Note that here the anti-TRIM32-1137 antibody has been used. (b) Immunostainings of satellite cells on myofibers from wild type mice cultured for the indicated time (left grey boxes) and labelled with the indicated markers (upper grey boxes). Note that here the anti-TRIM32 antibody M09 has been used. (c) Immunostainings of satellite cells on myofibers from wild type (+/+) and TRIM32−/− (−/−) mice labelled with the indicated markers (upper grey boxes). Note that here the anti-TRIM32 antibody 3150 has been used. (TIF) [file pone.0030445.s001.tif]

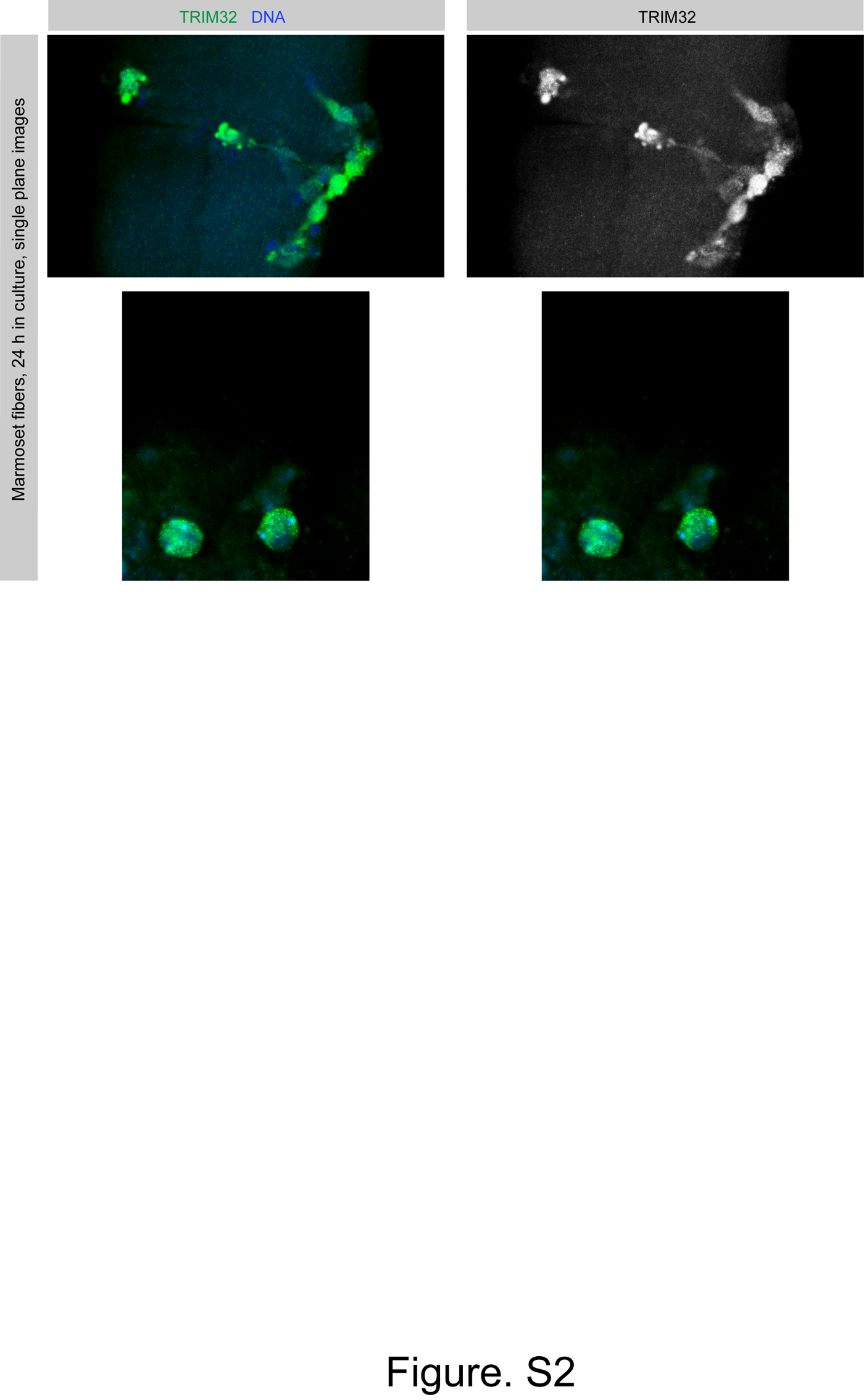

Supplement: Figure S2 — The expression pattern of TRIM32 is conserved in the Common Marmoset Callithrix jacchus . Immunostainings of satellite cells on Marmoset myofibers cultured for 24 h and labelled with the indicated markers (upper grey boxes). (TIF) [file pone.0030445.s002.tif]

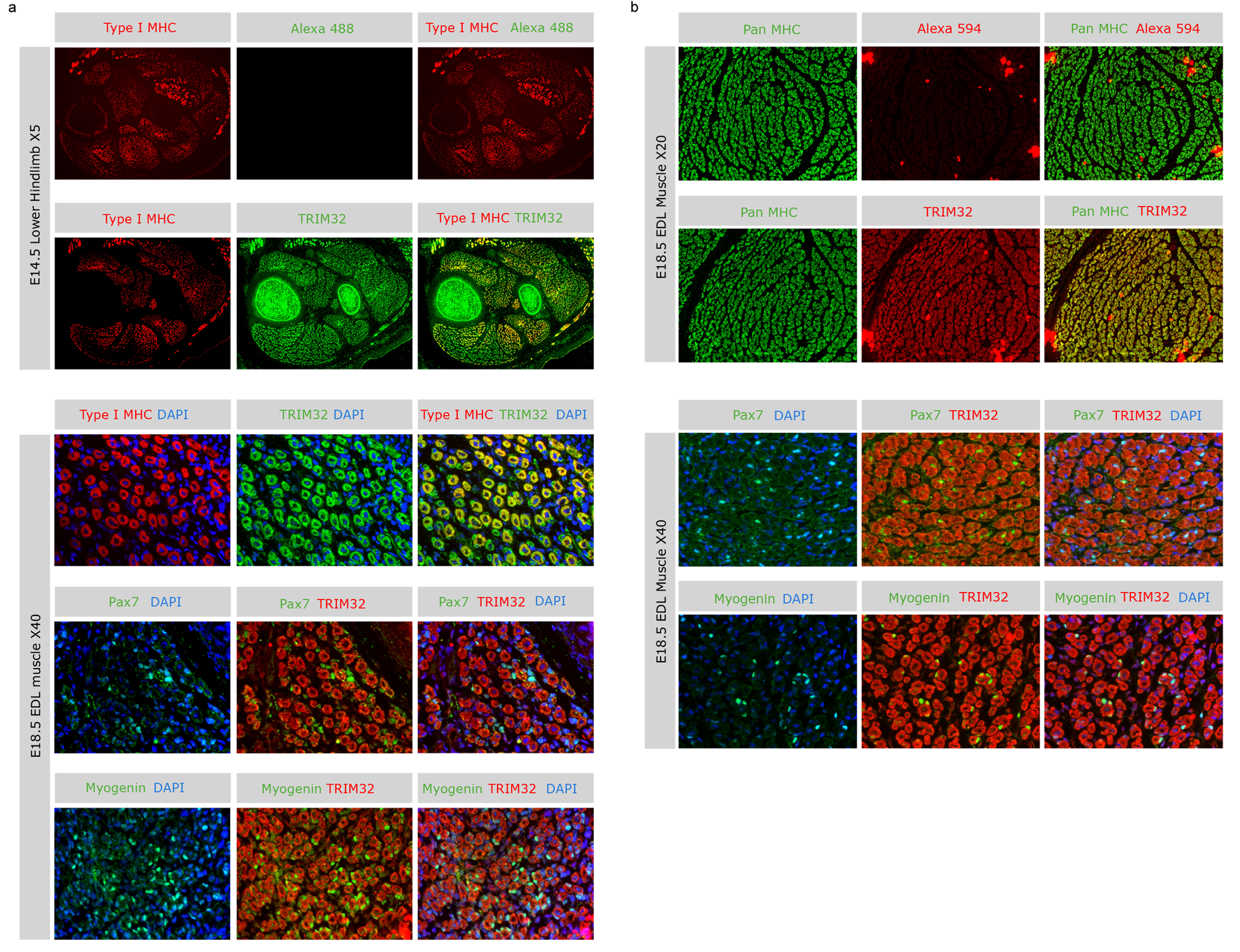

Supplement: Figure S3 — Expression of TRIM32 during muscle development. (a) Immunostainings of cross sections from wild type E14.5 Lower Hindlimb and E18.5 EDL Muscle labelled with the indicated markers (upper grey boxes). (b) Immunostainings of cross sections from wild type E18.5 EDL Muscle labelled with the indicated markers (upper grey boxes). (TIF) [file pone.0030445.s003.tif]

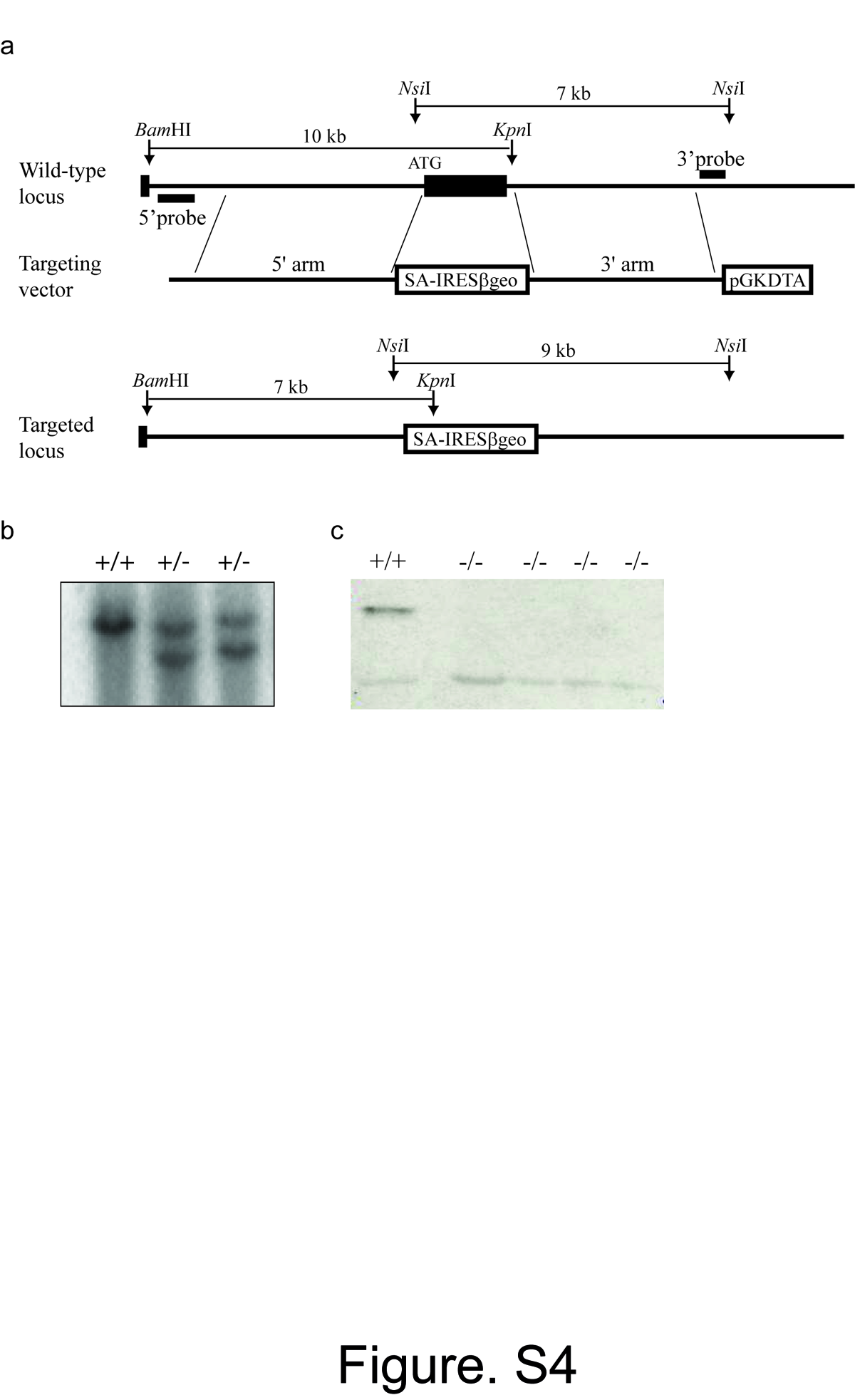

Supplement: Figure S4 — Gene-targeting strategy for creating the TRIM32 null allele. (a) Schematic representation of the TRIM32 genomic locus, the gene-targeting vector and the mutated locus with Exon 2 replaced by the SA-IRESβgeo cassette. (b) Southern Blot analysis of ES clones with insertion of targeting vector. Both 5′ and 3′ probes gave predicted digestion patterns. (c) Western Blot analysis of TRIM32 expression in cell lysates from mouse embryonic fibroblast derived from TRIM32 knock-out mice, demonstrating complete loss of TRIM32 expression in TRIM32−/− cells. (TIF) [file pone.0030445.s004.tif]

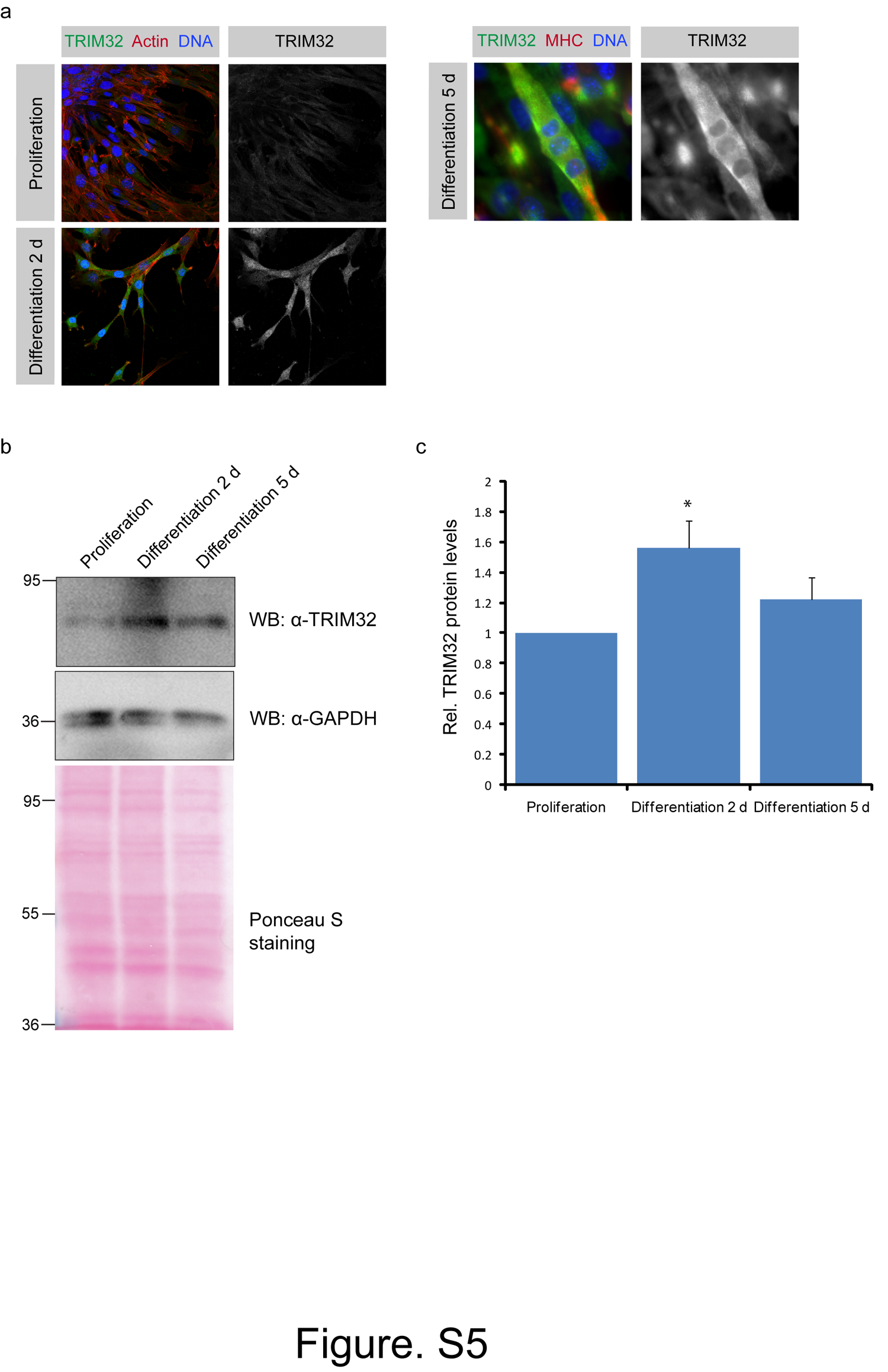

Supplement: Figure S5 — Expression of TRIM32 is upregulated during differentiation of C2C12 cells. (a) Immunostainings of C2C12 cells cultivated for 0 days, two days or 5 days under differentiation conditions and labelled with the indicated markers (upper grey boxes). (b) Western Blot analysis of TRIM32 expression in C2C12 cells cultivated for 0 days, two days or 5 days under differentiation conditions. (c) Diagram showing the relative levels of TRIM32 protein in C2C12 cells (Western Blot measurement as in (c)) cultivated under differentiation conditions. Ponceau S staining was used to normalize the protein levels. (TIF) [file pone.0030445.s005.tif]

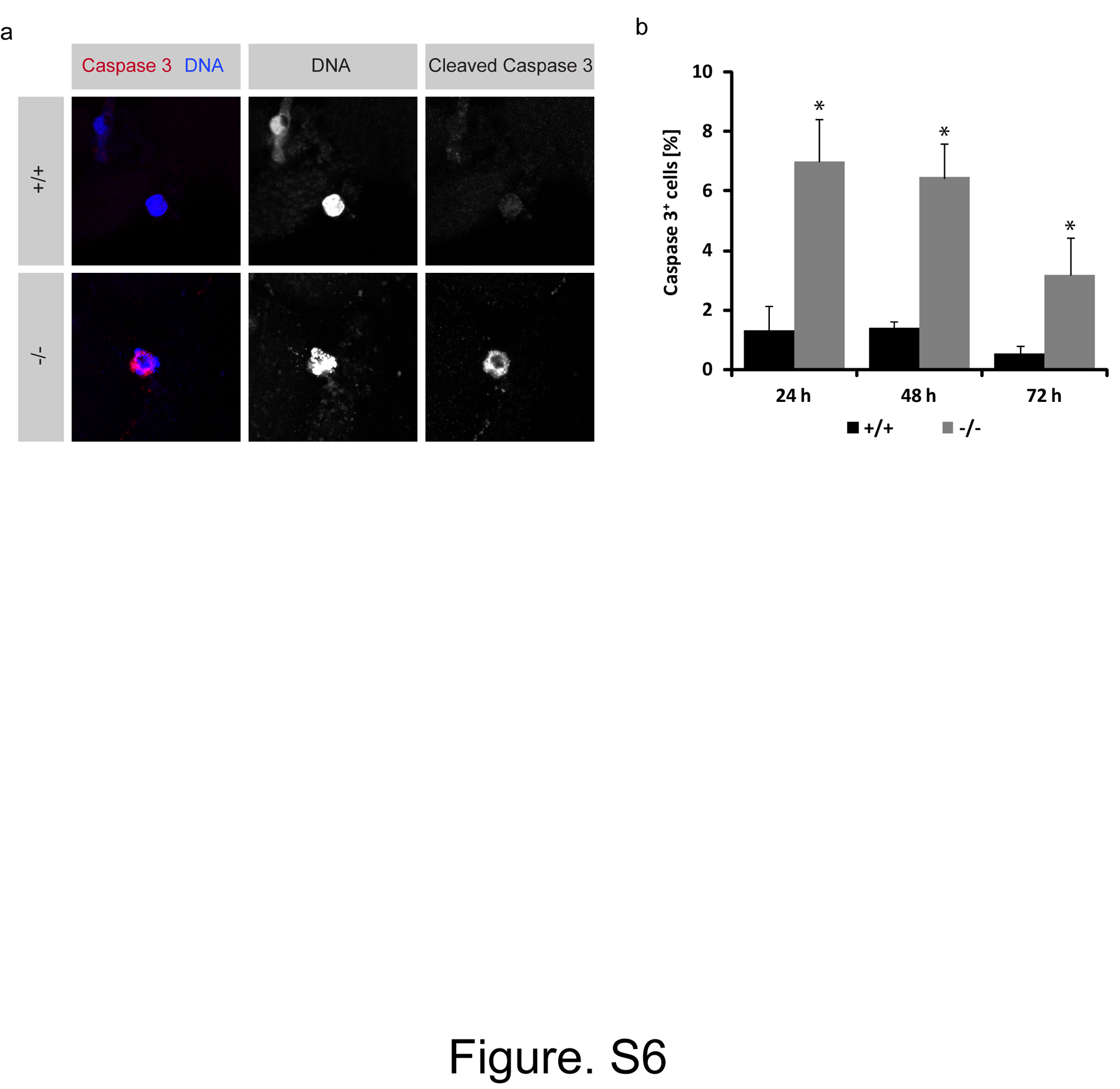

Supplement: Figure S6 — Increased apoptosis in satellite cells of TRIM32−/− mice. (a) Immunostainings of satellite cells on myofibers from wild type (+/+) and TRIM32−/− (−/−) mice cultured for 24 h and labelled with the indicated markers (upper grey boxes). (b) Diagram showing the fraction of satellite cells on myofibers of wild type (+/+) and TRIM32−/− (−/−) mice undergoing apoptosis (cleaved Caspase 3 positive) at 24 h, 48 h and 72 h.(mean ± std; *P<0.001 compared to wild type). (TIF) [file pone.0030445.s006.tif]

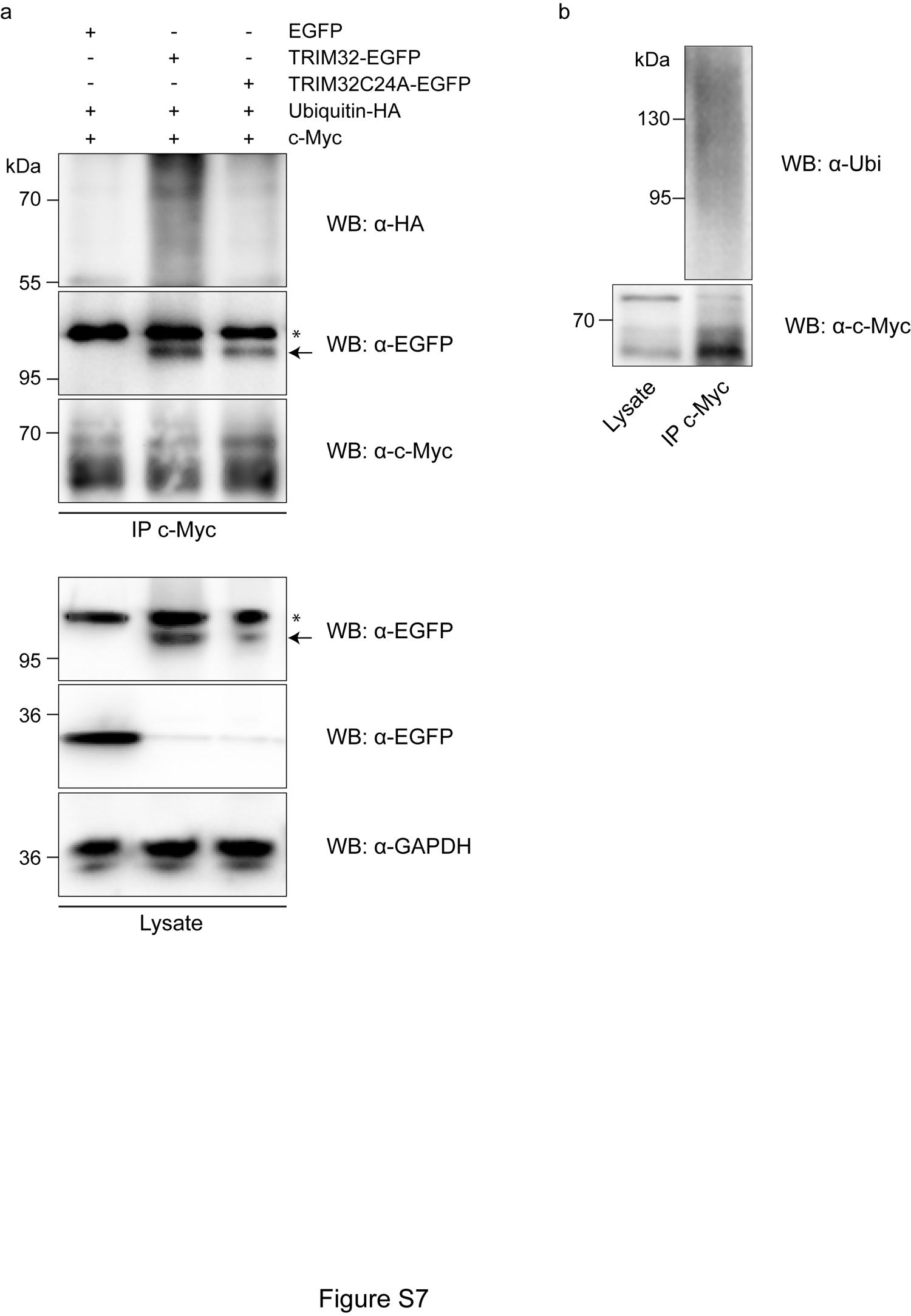

Supplement: Figure S7 — TRIM32 binds to and ubiquitinates c-Myc in C2C12 cells. (a) Western blot analysis of C2C12 cells transfected with the indicated constructs and immunoprecipitation of c-Myc with an anti-c-Myc antibody. Antibodies against c-Myc and HA were used for detection of ubiquitinated c-Myc after immunoprecipitation and an antibody against EGFP was used to detect the different TRIM32 constructs (arrow) that bound to c-Myc. In the lysate, the different TRIM32 constructs (arrow) and EGFP were detected with an anti-EGFP-antibody. Unspecific bands are indicated by asterisks. The GAPDH western blot is shown as loading control. (b) Western blot analysis of C2C12 cells transfected with HA-tagged Ubiquitin and cultivated for two days under differentiation conditions. The cells were treated for seven hours with the proteasome inhibitor MG-132 and c-Myc was immunoprecipitated using an anti-c-Myc antibody. Antibodies against c-Myc and mono- and poly-ubiquitinated proteins were used for detection of ubiquitinated c-Myc after immunoprecipitation. (TIF) [file pone.0030445.s007.tif]
